# Genetic Structure of Tibeto-Burman Populations of Bangladesh: Evaluating the Gene Flow along the Sides of Bay-of-Bengal

**DOI:** 10.1371/journal.pone.0075064

**Published:** 2013-10-09

**Authors:** Nurun Nahar Gazi, Rakesh Tamang, Vipin Kumar Singh, Ahmed Ferdous, Ajai Kumar Pathak, Mugdha Singh, Sharath Anugula, Pandichelvam Veeraiah, Subburaj Kadarkaraisamy, Brijesh Kumar Yadav, Alla G. Reddy, Deepa Selvi Rani, Syed Saleheen Qadri, Lalji Singh, Gyaneshwer Chaubey, Kumarasamy Thangaraj

**Affiliations:** 1 Center for Advanced Research in Physical, Chemical, Biological and Pharmaceutical Sciences, University of Dhaka, Dhaka, Bangladesh; 2 Centre for Cellular and Molecular Biology, Hyderabad, India; 3 Department of Evolutionary Biology, Institute of Molecular and Cell Biology, University of Tartu, Tartu, Estonia; 4 Estonian Biocentre, Tartu, Estonia; 5 Genome Foundation, Hyderabad, India; 6 Banaras Hindu University, Varanasi, India; University of Utah, United States of America

## Abstract

Human settlement and migrations along sides of Bay-of-Bengal have played a vital role in shaping the genetic landscape of Bangladesh, Eastern India and Southeast Asia. Bangladesh and Northeast India form the vital land bridge between the South and Southeast Asia. To reconstruct the population history of this region and to see whether this diverse region geographically acted as a corridor or barrier for human interaction between South Asia and Southeast Asia, we, for the first time analyzed high resolution uniparental (mtDNA and Y chromosome) and biparental autosomal genetic markers among aboriginal Bangladesh tribes currently speaking Tibeto-Burman language. All the three studied populations; Chakma, Marma and Tripura from Bangladesh showed strikingly high homogeneity among themselves and strong affinities to Northeast Indian Tibeto-Burman groups. However, they show substantially higher molecular diversity than Northeast Indian populations. Unlike Austroasiatic (Munda) speakers of India, we observed equal role of both males and females in shaping the Tibeto-Burman expansion in Southern Asia. Moreover, it is noteworthy that in admixture proportion, TB populations of Bangladesh carry substantially higher mainland Indian ancestry component than Northeast Indian Tibeto-Burmans. Largely similar expansion ages of two major paternal haplogroups (O2a and O3a3c), suggested that they arose before the differentiation of any language group and approximately at the same time. Contrary to the scenario proposed for colonization of Northeast India as male founder effect that occurred within the past 4,000 years, we suggest a significantly deep colonization of this region. Overall, our extensive analysis revealed that the population history of South Asian Tibeto-Burman speakers is more complex than it was suggested before.

## Introduction

**Figure 1:**
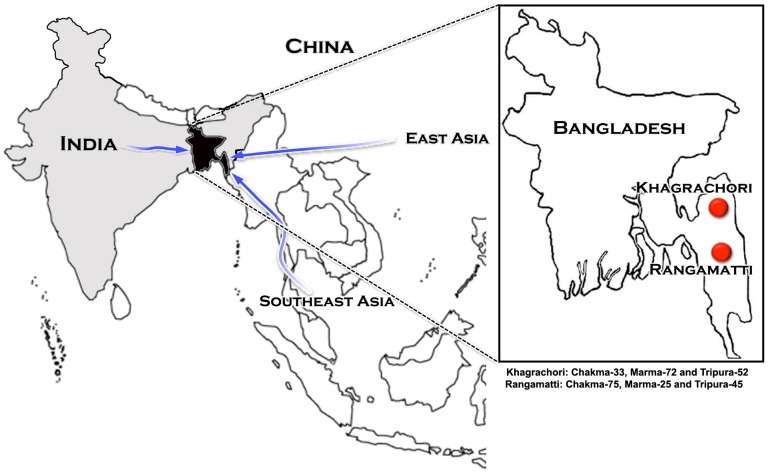
Map of Bangladesh showing the sampling area and suggested geneflow from different directions.

Bangladesh is bordered by Eastern India in west, Northeastern India in north and by the Bay of Bengal in the south. It also shares a narrow boundary with Myanmar on the southeastern rim (Fig. 1). Its geographical placement epitomized it as an important linguistic contact zone. There are 32 tribal populations documented in Bangladesh representing 1% of the total population [1]. The tribal populations living in the coastal as well as Chittagong Hill Tract regions are poorly understood. Gaps in our knowledge are substantial with respect to population ecologies, habitat and phylogenetic relationships. As a consequence, these tribal people haven't been studied at molecular resolution to determine their phylogenetic placement in terms of South Asia and East/Southeast (E/SE) Asian gene pool.

In the present study, we have carried out a detailed molecular analysis of three major tribal populations (Chakma, Marma and Tripura) living in Chittagong Hilly tract of Bangladesh. These populations are described as true aboriginal tribal populations of Bangladesh [2]. All the three populations analysed in this study speak a branch of Tibeto-Burman (TB) language. TB language family is considered as second largest language family in the world based on number of native speakers. In South Asia, TB language family is explicitly considered to have arrived from East and is mainly present in Nepal, Bhutan, Bangladesh, Northeast India and in some parts of Pakistan; where the Balti population from the Karakoram Mountains speaks a TB branch [3]. It has been speculated that the TB language family originated in China and spread from the Yellow river of China into Myanmar and the greater Himalayan region [3]. The overwhelming majority of languages spoken in the greater Himalayan fringes belong to this language family. The internal classification of TB is still controversial [4] and needs a thorough vocabulary and lexical reconstructions. Genetic studies on limited populations of this group however, suggests their recent migration from East to Indian subcontinent [5]–[7].

The aim of this study was to trace footprints of initial Paleolithic colonization and the much later Neolithic expansion of populations among the aboriginal tribal populations living in Chittagong Hill Tract region of Bangladesh. We also assessed whether South Asian TB speaker's genetic variation was structured by the language or by their geographical placement. We also examined the relationships of the studied populations with Indian, Southeast Asian TB and other linguistic groups.

## Results and Discussion

### Highly permeable maternal boundaries

Marma and Tripura populations share high frequency of Indian and low frequency of East Asian specific maternal haplogroups, than Chakma (Table 1). East Asian specific Haplogroup F1 was observed highest among all the studied populations (Table 1 and Table S1), and shared their closest haplotypes with Northeast Indian populations. In contrast to the high endogamy observed among other Indian populations [8]–[12], the massive haplogroup sharing among all the three populations suggest that the maternal boundaries in this region is highly permeable (Table 1).

**Table 1:** mtDNA and Y chromosome haplogroup frequencies among the tribal populations of Bangladesh.

mtDNA																			
Population	*n*	A	A10	B4	C1	D4	D5	E	F1	G2a	G3	M	M10	M12	M13	M18	M2	M20	
Chakma	**108**	0.01	0.01	-	-	0.03	0.05	-	0.20	0.02	0.01	0.07	-	0.02	0.04	0.01	0.02	0.04	
Marma	**97**	0.02	-	0.01	0.01	-	-	-	0.12	0.06	0.01	0.11	0.03	-	0.05	-	-	0.12	
Tripura	**97**	0.01	-	-	-	-	-	0.02	0.24	-	0.01	0.12	0.01	-	0.04	0.01	-	0.03	

Population	*n*	M25	M3	M33	M33c	M4'67	M47	M48	M49a	M49c	M5	M51	M5a	M60	M61	M62	M7	M72	
Chakma	**108**	0.01	-	-	-	-	-	-	-	0.01	0.02	0.01	0.02	-	0.02	-	0.01	-	
Marma	**97**	0.02	-	0.01	0.01	0.01	0.01	-	0.05	-	-	-	0.02	0.01	-	-	-	-	
Tripura	**97**	-	0.05	-	0.01	-	-	0.01	0.08	0.02	0.01	-	0.01	-	-	0.01	-	0.02	

Population	*n*	M74	M75	M7b	M7c3a	M8	M8a	M9a	N21	R	R22	R3	R31a	R9b	Z	U	U7	W	Hgp. Div.
Chakma	**108**	0.07	-	0.01	0.06	0.03	-	0.04	-	0.04	0.06	-	0.03	0.04	0.03	-	-	-	0.9858
Marma	**97**	-	-	0.06	0.06	-	0.02	-	-	0.01	0.06	-	-	0.02	0.04	0.01	-	0.01	0.9933
Tripura	**97**	0.03	0.01	0.01	0.05	-	0.01	0.03	0.01	0.01	0.03	0.01	0.02	0.03	-	-	0.02	-	0.9871

Y chromosome																			
Population	*n*	C	D	F	J2	K*	L	NO	N1a	O	O2a	O3	O3a3c	P	R1	R1a1	R2	Hgp. Div.	
Chakma	**89**	0.01	0.01	0.02	0.01	-	-	-	0.07	0.06	0.16	0.01	0.42	0.15	0.07	0.01	0.01	0.7763	
Marma	**60**	0.03	0.02	-	-	0.02	0.05	0.02	-	0.03	0.17	0.03	0.50	0.07	-	0.03	0.03	0.7232	
Tripura	**88**	0.03	0.01	0.03	0.02	0.02	0.07	-	0.05	-	0.18	0.09	0.35	0.07	0.03	-	0.03	0.8268	

The haplotype based comparison with neighboring Indian and Southeast Asian populations showed a closest match with Northeast India, implying their close genetic connection (Table S1). The haplotype diversity is highest among Marma, followed by Tripura and Chakma (Table 1). To see whether the Indian or the East Asian-specific haplogroups have higher diversity in this region, we have calculated the haplotype diversity of the HVS-I haplotypes from the aggregate data of the three populations. Indian-specific haplogroups have moderately higher haplotype diversity 0.989 (95% CI: 0.995, 0.983) than the East Asian-specific haplogroups 0.972 (95% CI: 0.975, 0.969), suggesting that the Indian-specific haplogroups were likely to be present before the geneflow from the East. However, high haplotype diversity may not always be a reflection of older lineages [13], and as this region share land borders from Northeastern as well as Eastern India, with a caution it could also be a consequence of repeated gene flow from two diverse sources and same explanation also applies to the East Asian maternal diversity of this region.

**Figure 2:**
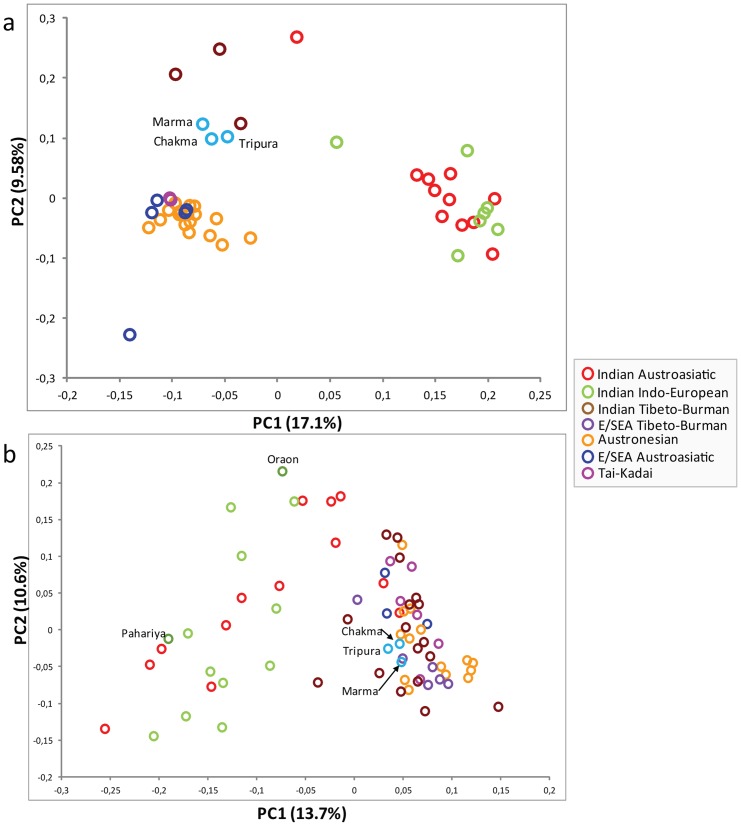
PCA plots constructed on the basis of (a) mtDNA haplogroup frequencies and (b) Y chromosome haplogroup frequencies.

To learn more about the maternal relation of studied tribal populations with the neighboring populations, we performed Principal Component Analysis (PCA). PC1 was basically segregating Indian and E/SE Asian populations, while PC2 was setting apart loosely packed Indian and TB populations of Bangladesh from E/SE Asians and Indian groups (Fig. 2a). All the studied populations cluster tightly with one another and loosely with other Indian TB populations. In a geographical comparison, the Tibeto-Burman populations of Bangladesh remained closer to E/SE Asian cluster than Indian cluster.

### Paternal Ancestry of Bangladeshi tribal populations

To gain insight into their paternal gene pool, we have analyzed Y chromosomal data from 89 Chakma, 60 Marma and 88 Tripura individuals. Over 35 biallelic markers and 17 short tandem repeats (STRs) loci were genotyped to produce a high-resolution dataset of Y chromosomes from these groups. We observed considerable differences in Indian specific NRY haplogroup frequencies among the three groups, Chakma has the least, while Tripura has the highest Indian-specific haplogroups (Table 1). More than 60% of the individuals from all the three tribal populations are skewed towards Southeast Asian-specific haplogroups O2 and O3 (Table 1). All populations had high to moderate frequencies of C, D, O2a1, O3, P and R2 haplogroups.

Notably, none of the studied populations carry Indian-specific Paleolithic haplogroup H or any of its branches (Fig. S1), which is also reported in East and Southeast Asia among Balinese, Tibetans, Mongolians, Cambodians and Laosians [14,–17]. Unlike haplogroup H, haplogroup P that has unknown origin is substantially present among all the three studied populations (Table 1), and also reported in the adjoining regions [18]. A detailed phylogeographic study of haplogroup P would throw more light about its origin and expansion in this region. In spite of sharing a larger gene pool with TB populations of Northeast India, in the wider geographical context, the paternal haplogroup frequency distribution among TB tribes of Bangladesh exhibits a unique trend of haplogroup composition, e.g., the occurrence of Indian specific haplogroups L and E/SE Asian specific haplogroup NO, which are completely absent among Northeast Indian populations (Fig. S1), thus indicating geneflow from three distinct directions (Fig. 1).

Similar to mtDNA PCA, in Y chromosome haplogroup based PCA, PC1 differentiates Indian with E/SE Asian clusters, while, PC2 sets apart Indian-Indian and E/SE Asian-E/SE Asian populations (Fig. 2b). The Bangladeshi TB populations cluster together with E/SE Asian specific group along with Indian TB speakers. This is due to overwhelming majority of East Asian-specific haplogroups in these populations (Table 1). Indian Indo-European and Dravidian populations follow their geographical placement in the plot rather than their language affiliation. Unlike the Munda speakers of India where male mediated migration from Southeast Asia shaped the current landscape of their gene pool, among South Asian TB the presence of frequent East Asian maternal and paternal haplogroups suggest that both males and females played largely equal roles in TB expansion in Southern Asia.

To assess whether geography or linguistic affiliation was a better predictor of genetic variation, we conducted AMOVA analysis (Table 2). We noted that the variation among groups was not significant, and among population within-group variation was high. When the data were clustered by linguistic affiliation, among group variation rose to 18%, whereas the among population within-group value fell to about 4% (Table 2). This finding suggested that their paternal genetic history is structured along linguistic instead of geographic lines.

**Table 2:** Analysis of molecular variance (AMOVA).

Grouping (number of groups)	Among groups	Among populations within groups	Within populations
Language (5)	17.9	3.8	78.3
Geography (5)	3.1	29.3	67.6

The language groups used for the analysis were Indo-European, Dravidian, Austroasiatic, Tibeto-Burman and Austronesian, while, geography was divided in to East Asia, Southeast Asia, Northeast India, East Bengal and West Bengal. All p-values were significant (P<0.05) except for the Among groups-geography (3.1; p = 0.12).

### High Diversity and Deep coalescent expansion ages of founder haplogroups

Because variation accumulates with time, a relative chronology can be constructed by assessing haplogroup STR variance, haplotype diversity (HD), ρ (rho), and mean pairwise differences (MPD) (Table 3, and Tables S2 and S3). We noted the highest diversity of haplogroup O2a1 than N1a, NO and O3a2c1 haplogroups. Consistently Chakma had greater diversity than Marma and Tripura (Table 3). In the NETWORK analysis of 15 STRs, most of the haplotypes were unique and dispersed among longer branches of network, except three pairs of shared haplotypes of Tripura (Fig. S2).

**Table 3:** Y-STR Diversity estimates of major haplogroups.

Haplogroup	Population(s)	*n*	*h*	HD	MPD	Variance	Age (Kya)	SD (Kya)
N1a	Chakma	4	4	1.000±0.177	7.500±4.441	0.35	11.47	2.42
N1a	Tripura	2	2	1.000±0.500	5.000±3.873	0.27	4.83	2.48
**N1a**	**Chakma+Tripura**	**6**	**6**	**1.000**±**0.096**	**6.467**±**3.567**	**0.30**	**11.67**	**2.92**
**NO**	**Chakma+Marma+Tripura**	**3**	**3**	**1.000**±**0.272**	**7.667**±**4.928**	**0.29**	**9.66**	**1.75**
O2a	Chakma	6	6	1.000±0.096	8.400±4.537	0.37	12.68	1.54
O2a	Marma	9	9	1.000±0.052	7.556±3.896	0.35	15.82	2.65
O2a	Tripura	10	10	1.000±0.045	7.889±4.011	0.38	15.22	2.52
**O2a**	**Chakma+Marma+Tripura**	**25**	**25**	**1.000**±**0.011**	**7.717**±**3.720**	**0.38**	**16.23**	**2.01**
O3a3c	Chakma	16	16	1.000±0.022	7.192±3.558	0.35	15.55	2.18
O3a3c	Marma	14	14	1.000±0.027	6.693±3.359	0.30	13.11	2.28
O3a3c	Tripura	15	12	0.971±0.033	6.715±3.355	0.35	15.30	2.84
**O3a3c**	**Chakma+Marma+Tripura**	**45**	**42**	**0.997**±**0.006**	**7.188**±**3.4328**	**0.38**	**15.94**	**2.14**

**Figure 3:**
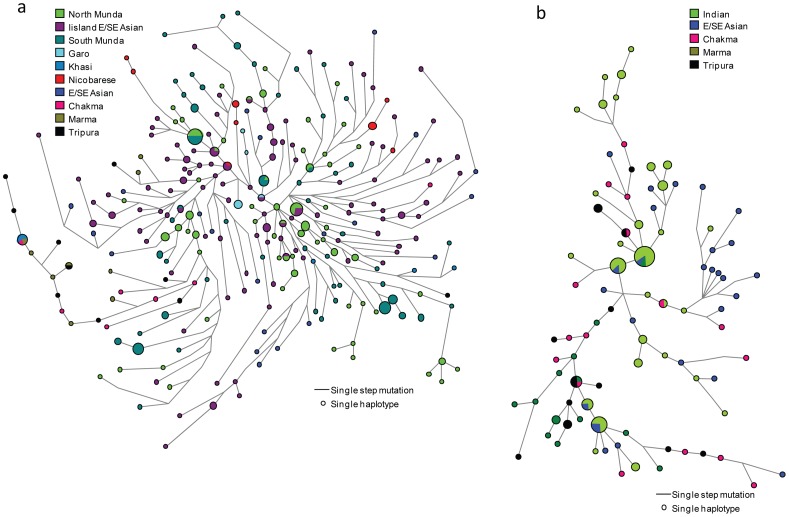
Unrooted phylogenetic network of a) haplogroups M95-O2a1; b) haplogroup M134-O3a2c1. The network was constructed using a median-joining algorithm as implemented in the Network 4.6.0 program. The size of the circles is proportional to the number of samples.

To compare them with the surrounding populations, we have redone NETWORK analysis of O2a1 and O3a2c1 haplogroups based upon common STRs from published sources [14], [19]. In haplogroup O2a1, the Bangladeshi TB populations form their own cluster and share some of their haplotypes with the Khasi population of Meghalaya, which speaks Khasi-Asalian branch of Austroasiatic language (Fig. 3a). The sharing of common haplotypes with Khasi advocates a recent geneflow beyond the borders of language. For a wider geographical coverage, we pulled all Bangladeshi TBs into a single group and reduced the number of common STRs to eight (Fig. S3). All the Bangladeshi individuals form their own cluster with Indian TB populations rooting out from Southeast Asian Island haplotype. In haplogroup O3a2c1, most of the Bangladeshi TB populations largely allocate their branches or haplotypes with Indian TB haplotypes (Fig. 3b).

The coalescent ages of haplogroups O2a1 and O3a2c1 were almost similar ranging between 14 and 18 Kya (Table S2), suggested that they arose before the differentiation of any language group and approximately at the same time. Contrary to the scenario proposed for colonization of northeast India as male founder effect have occurred within the past 4,000 years [6], we suggest a significantly deep colonization of this region. STR haplotypes of haplogroups O2a1 and O3a2c1 showed the greatest variance and age estimates among Southeast Asians, moderate values among Bangladeshi aboriginal tribal and the lowest values in the Indians (Table S2), thus forming a east to west gradient of O2a1 and O3a2c1 diversity and determine the directionality of movement by bearers of O2a1 and O3a2c1 Y chromosomes. This trend of slightly decreasing values from east to west suggests an origin for O2a1 and O3a2c1 in the Southeast Asia and its dispersal through an westward expansion minimum with two different waves – first the Austroasiatic speakers carrying O2a1 (likely without O3 lineage) and second Tibeto-Burman containing O3 and O2a1 lineages.

### Higher Indian ancestry component than Northeast Indian populations

**Figure 4:**
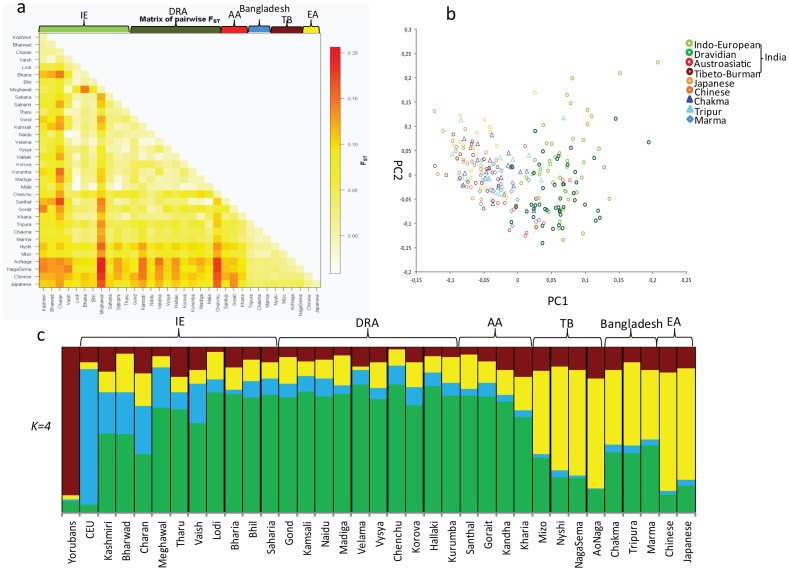
Autosomal Analysis. a) Heat map of pairwise F*st* of Bangladeshi tribal groups, in comparison with Indian and East Asian populations; b) PCA of Indian, East Asian and Bangladeshi groups. PC analysis was carried out using smartpca program (with default settings) of the EIGENSOFT package, PC1 represent 2.1%, while PC2 shows 1.7% of variations; c) Bar plot displays individual ancestry estimates for studied populations from a structure analysis by using STRUCTURE with *K* = *4*.

To see the population structure and ancestry components among the studied population and surrounding groups, we have studied previously used 50 Ancestry Informative Markers (AIMs) [12], [20] and combined them with the same numbers of markers extracted from the literature [21], [22]. Four markers failed from the 99% quality control, therefore, we have used 46 AIMs for further analysis. The pairwise F*st* among studied population showed a consistent pattern for all the studied loci (Table 4). Chakma and Tripura share the maximum genetic similarity while, Marma and Tripura were having highest F*st* value. The Bangladeshi TB populations showed a closer affinity with Northeast Indian as well as East Asian populations (Fig. 4a and Table S4). The PCA with Indian and East Asian populations placed the Bangladeshi and Indian TB populations together with East Asians, however, there were some individuals of Tripura and Marma falling in India cluster (Fig. 4b). The STRUCTURE analysis of the studied populations in the context of rest of the world showed two major shared components among the studied populations (Fig. 4c). First shared component was higher among Indian populations, while the second was specific to East Asians. It was intriguing that the Bangladeshi TB populations were carrying substantially higher Indian ancestry component than the Northeast Indian TB populations, suggesting that the primary genetic structure of Bangladeshi populations was likely made up of the similar gene pool as Indian, which was further electroplated by the TB expansion.

**Table 4:** Mean pairwise F*st* values for various loci between studied populations.

	mtDNA	Y chromosome	Autosomes
**Chakma Vs. Marma**	0.0095	0.0019	0.0035
**Chakma Vs. Tripura**	0.0051	0	0.0008
**Marma Vs. Tripura**	0.0155	0.0033	0.0104

In conclusions, our extensive analysis on uniparental and biparental markers led to the more precise identification of Indian and E/SE Asian haplogroups among Bangladeshi aboriginal tribal populations and a better understanding of the extent of ancient and recent admixtures. We present substantial evidence for multiple genetic strata that arose likely through a series of distinct migratory processes. In contrast to Indian context, where gene is highly correlated with geography, we found that the Northeast Indian and Bangladeshi landscape is associated with language. The post-Neolithic incursions make a major impact on the composite gene pool of the studied tribal populations, consistent with the genetic impact of the language. In addition, by generating well resolved mtDNA and Y chromosomal lineages, we were able to detect the directional geneflow and their conglomerisation from east and west of Bangladesh.

## Methods

### Sampling

About 5 ml blood samples were collected from each individual belonging to three tribes (108 Chakma, 97 Marma and 97 Tripura) from Rangamatti and Khagrachori regions of Chittagong Hill Tract in Bangladesh. All the three populations are contributed by these two places together (Fig. 1). These are major tribal groups inhabited in Bangladesh and represent approximately 90% of the total tribal population of Bangladesh. The sampling was done with an interview protocol that involved the queries pertaining to name of the tribe which they belonged, place of their ancestor, any oral history about their ancestry, etc. The related individuals with similar family background were avoided. This project was approved by the Institutional Ethical Committee of the Centre for Advanced Research in Sciences, University of Dhaka, Bangladesh and the Centre for Cellular and Molecular Biology-CSIR, Hyderabad, India. The informed written consent was obtained from all the volunteers who participated in this study.

### Genotyping

Over 32 Y chromosome biallelic markers from previously published dataset [23] were used in this study for assigning the haplogroup to each individual. A battery of 17 Y-STRs loci were amplified using Y-filer® kit (Applied Biosystems, Foster City, USA) in reaction volumes of 10 µl with 1 U of AmpliTaq Gold® DNA polymerase (Applied Biosystems, Foster City, USA) with the manufacturer protocol. The PCR amplicons along with GS500 LIZ (as size standard) were analysed in the ABI 3730 DNA Analyzer (Applied Biosystems, Foster City, USA). GeneMapper v4.0 software program (Applied Biosystems, Foster City, US) was used to analyze the raw data.

We sequenced the hypervariable segment I (HVS-I) of mtDNA and the variations were scored against the Reconstructed Sapiens Reference Sequence (RSRS) [24]. Haplogroups were assigned based on HVS-I variations and are further confirmed by genotyping the coding region mutations published till date in PhyloTree (www.phylotree.org.). Along with Y chromosome and mtDNA markers, 50 AIMs published elsewhere [20], were also genotyped to infer the ancestry of Bangladesh population.

### Statistical analysis

Fragment sizes of Y-STRs, were determined using the GeneMapper® Analysis Software v4.0 and allele designations were based on comparison with allelic ladders provided by the manufacturer. Out of 17 loci obtained, two DYS385 loci were excluded from the current analyses because they could not be distinguished using the typing method employed. DYS 389I (DYS 389cd) was subtracted from DYS389II and re-named DYS389ab. Thus, all the analysis linked with Y-STRs data were carried out with 15 loci. A median-joining network, resolved with the MP algorithm, was constructed using the Network package (version 4.5.0.2) (www.fluxus-engineering.com). The age of O2a1-M95 and O3a2c1- M134 was estimated from microsatellite variation within the haplogroup using the method described by Zhivotovsky et al. [25] and updated in Sengupta et al. [14].

Principal component analysis (PCA) for mtDNA and Y chromosomal haplogroups was performed using POPSTR, kindly provided by H. Harpending, and for autosomal SNP we used smartpca programme [26]. The analysis of molecular variance (AMOVA) was performed using Arlequin 3.5 [27]. For autosomal data, we filtered the combined data sets by using PLINK software 1.07 [28] to include only SNPs with a minor allele frequency >1% and genotyping success >99%. We calculated mean pairwise F*st* values between populations for all autosomal SNPs using the approach of Cockerham and Weir [29] We ran STRUCTURE [30] for the pruned data set (46 SNPs) from *K* = 2 to *K* = 8 (25 runs at each *K*) and selected the *K* (K = 4) that maximizes the posterior probability of the data, as explained by the developers [31] All structure runs performed 40,000 iterations after a burn-in of 50,000, following the default settings i.e. the admixture model, and assumed that allele frequencies were correlated.

## Supporting Information

Figure S1 Y chromosome haplogroups observed among the studied populations with major Y chromosome biallelic markers.
(TIF)

Figure S2 Unrooted phylogenetic network of a M214 derived individuals.
(TIF)

Figure S3 Unrooted phylogenetic network Tree of all Bangladeshi Tibeto-Burmans with eight common Y-STRs.
(TIF)

Table S1 mtDNA variations observed among the studied populations.
(DOC)

Table S2 Y-chromosome age estimates for haplogroups O2a and O3a3c among population groups of India E/SE Asia and Bangladesh.
(DOC)

Table S3 Y STR haplotype data of the studied populations.
(DOC)

Table S4 Pairwise population Fst values for the autosomal analysis.
(XLS)
