# Supplementary figures and images for: Genetic Structure of Tibeto-Burman Populations of Bangladesh: Evaluating the Gene Flow along the Sides of Bay-of-Bengal
Source: PLoS One. 2013 Oct 9;8(10):e75064. doi: 10.1371/journal.pone.0075064 (PMC3794028; doi:10.1371/journal.pone.0075064)

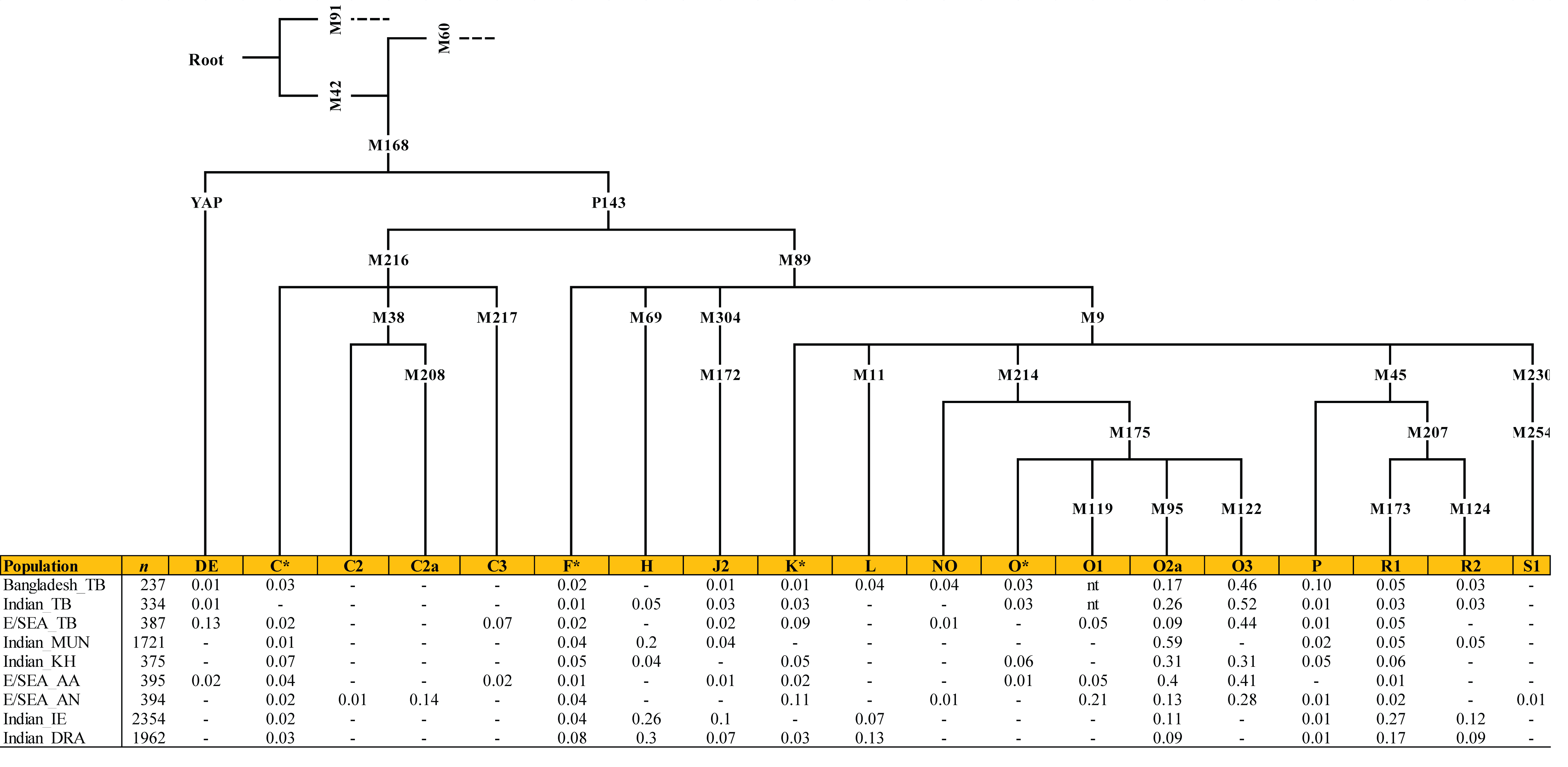

Supplement: Figure S1 — Y chromosome haplogroups observed among the studied populations with major Y chromosome biallelic markers. (TIF) [file pone.0075064.s001.tif]

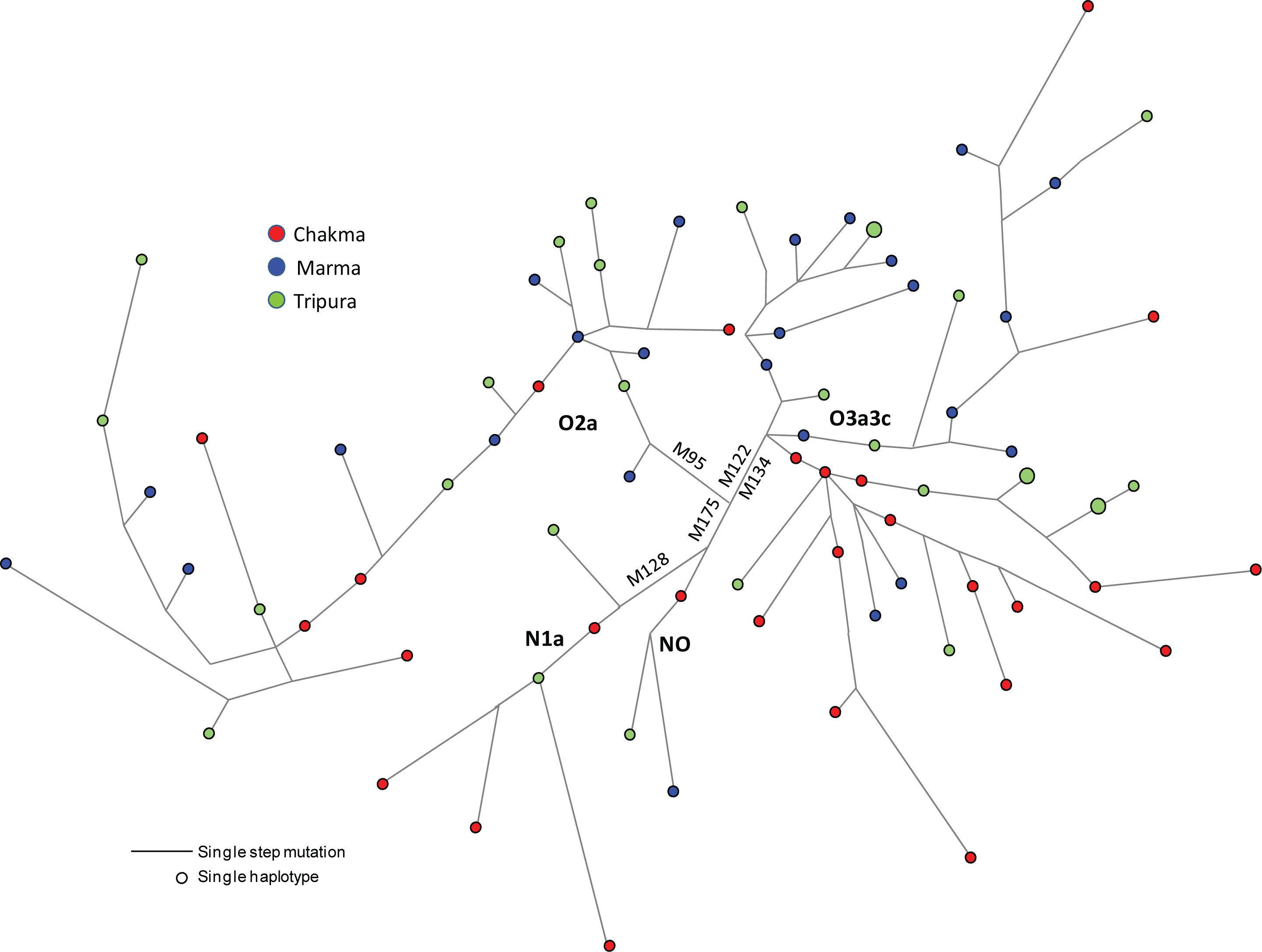

Supplement: Figure S2 — Unrooted phylogenetic network of a M214 derived individuals. (TIF) [file pone.0075064.s002.tif]

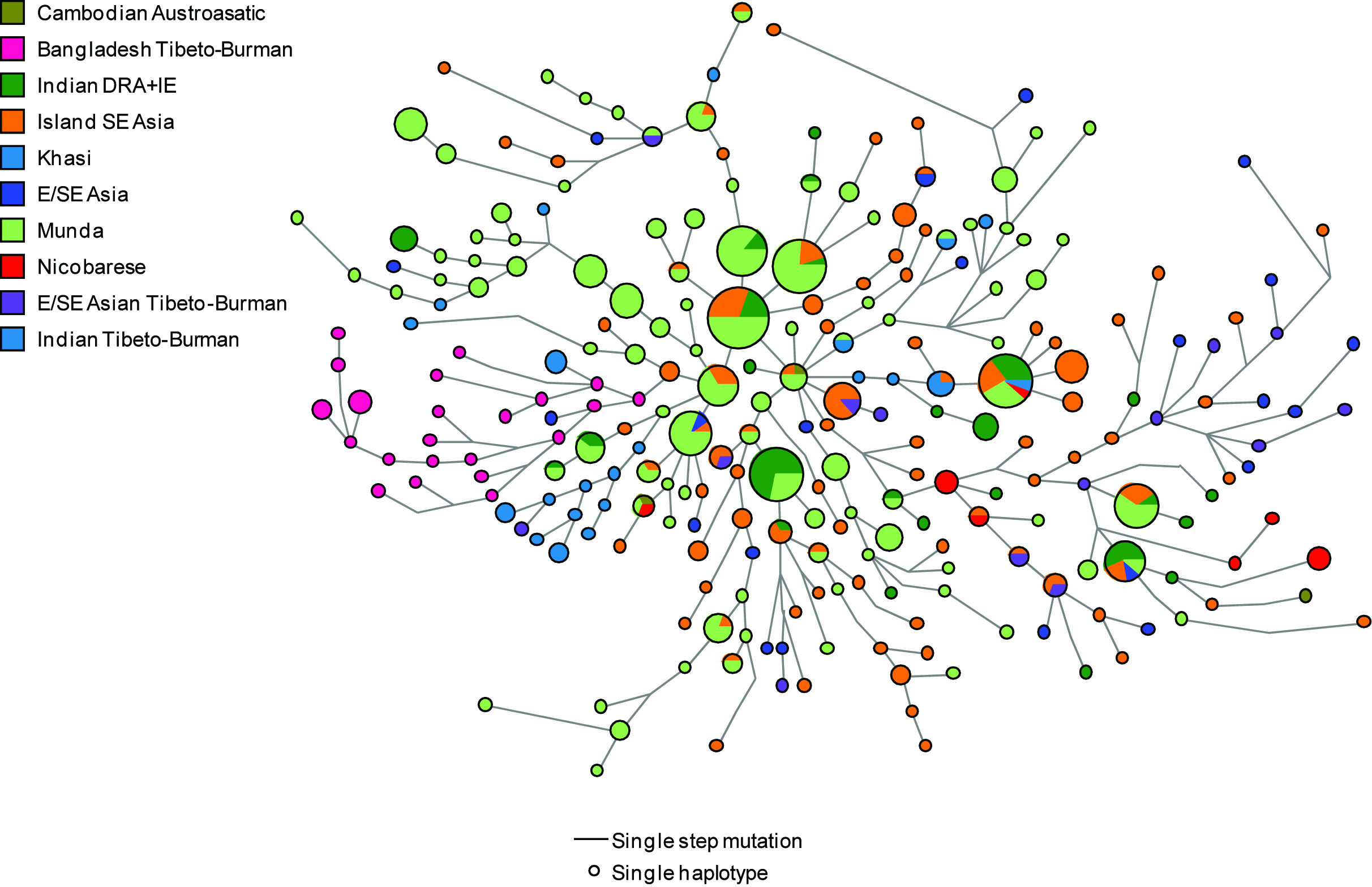

Supplement: Figure S3 — Unrooted phylogenetic network Tree of all Bangladeshi Tibeto-Burmans with eight common Y-STRs. (TIF) [file pone.0075064.s003.tif]
