# Supplementary material for: Genetic Structure of Tibeto-Burman Populations of Bangladesh: Evaluating the Gene Flow along the Sides of Bay-of-Bengal
Source: PLoS One. 2013 Oct 9;8(10):e75064. doi: 10.1371/journal.pone.0075064 (PMC3794028; doi:10.1371/journal.pone.0075064)
Supplement: Table S1 — mtDNA variations observed among the studied populations. (DOC) [file pone.0075064.s004.doc]

| **Sample ID** | **Population** | **HVSI (16000-16525bp)** | **HG.** | **Other mutations** |
| --- | --- | --- | --- | --- |
| B-2166 | Chakma | 86-172-223-362 | **D5** | 3873-10397-10398-10400 |
| C_212 | Chakma | 189 | **R** | 15301 |
| C-183 | Chakma | 129-223-290-319 | **A** | 663 |
| C-184 | Chakma | 111-129-304 | **F1** | 249d-3398-10310-12406 |
| C-185 | Chakma | 111-129-304 | **F1** | 249d-3398-10310-12406 |
| C-186 | Chakma | 223-381-390 | **M13** | 152-10398-10400-15924 |
| C-187 | Chakma | 86-129-209-223-272 | **M20** | 249d-3200-10398-10400-15691 |
| C-188 | Chakma | 86-129-209-223-272 | **M20** | 249d-3200-10398-10400-15691 |
| C-189 | Chakma | 192-223-249-319-381 | **M7c3a** | 5442-9824-10398-10400 |
| C-192 | Chakma | 129-162-172-293-304 | **F1** | 249d-10310-12406 |
| C-193 | Chakma | 93-129-223-248 | **M5** | 1888-10398-10400 |
| C-195 | Chakma | 136-189-223-243 | **M** | 10398-10400-15440 |
| C-196 | Chakma | 136-189-223-284 | **M** | 10398-10400-15440 |
| C-200 | Chakma | 75-93-129-223-248-291-399 | **M5a** | 709-1888-10398-10400 |
| C-201 | Chakma | 129-162-172-304 | **F1** | 249d-10310-12406 |
| C-202 | Chakma | 86-129-223-272 | **M20** | 249d-3200-10398-10400-15691 |
| C-203 | Chakma | 185-223-260-298-519 | **Z** | 10398-10400-15784 |
| C-204 | Chakma | 185-223-260-519 | **Z** | 10398-10400-15784 |
| C-205 | Chakma | 111-168-172-189 | **R31a** | 146-338-15884 |
| C-206 | Chakma | 111-168-172-183-189 | **R31a** | 146-338-15884 |
| C-207 | Chakma | 111-168-172-189 | **R31a** | 146-338-15884 |
| C-208 | Chakma | 111-129-189-304 | **F1** | 249d-10310-12406 |
| C-209 | Chakma | 129-213-362 | **R** | 15301 |
| C-213 | Chakma | 86-223-290-362 | **D4** | 3010-8414-10398-10400-10646 |
| C-214 | Chakma | 86-223-362 | **D4** | 3010-8414-10398-10400 |
| C-215 | Chakma | 111-129-172-304 | **F1** | 249d-10310-12406 |
| C-217 | Chakma | 172-223-311-362 | **M74** | 8251-10398-10268-10400 |
| C-218 | Chakma | 150-185-223-260-298 | **Z** | 10398-10400-15784 |
| C-220 | Chakma | 223-239-298 | **M8** | 8584-10398-10400 |
| C-221 | Chakma | 223-239-298 | **M8** | 8584-10398-10400 |
| C-222 | Chakma | 86-223-239-298-309 | **M8** | 8584-10398-10400 |
| C-224 | Chakma | 223-311-362 | **M74** | 8251-10398-10268-10400 |
| C-225 | Chakma | 172-223-311-362 | **M74** | 8251-10398-10268-10400 |
| C-226 | Chakma | 093-111-192-223-249-381 | **M7c3a** | 5442-9824-10398-10400 |
| C-227 | Chakma | 223-381-390 | **M13** | 152-10398-10400-15924 |
| C-228 | Chakma | 93-129-223-240-284-290 | **M12** | 3666-10398-10400-15010 |
| C-229 | Chakma | 129-145-249-288-301-304-311 | **R22** | 152-329 |
| C-230 | Chakma | 129-145-249-288-301-304-311 | **R22** | 152-329 |
| C-232 | Chakma | 93-129-223-240-248-290 | **M12** | 3666-10398-10400-15010 |
| C-233 | Chakma | 223-381-390 | **M13** | 152-10398-10400-15924 |
| C-234 | Chakma | 223-381-390 | **M13** | 152-10398-10400-15924 |
| C-235 | Chakma | 129-145-234-249-288-301-304 | **R22** | 152-329 |
| C-236 | Chakma | 218-289-304-526 | **R** | 15301 |
| C-237 | Chakma | 111-129-304 | **F1** | 249d-3398-10310-12406 |
| C-238 | Chakma | 129-145-249-288-301-304-311 | **R22** | 152-329 |
| C-239 | Chakma | 129-162-172-304 | **F1** | 249d-10310-12406 |
| C-240 | Chakma | 129-145-249-288-301-304-311 | **R22** | 152-329 |
| C-241 | Chakma | 129-162-172-304 | **F1** | 249d-10310-12406 |
| C-241a | Chakma | 129-145-249-288-301-304-311 | **R22** | 152-329 |
| C-242 | Chakma | 223-311-362 | **M74** | 8251-10398-10268-10400 |
| C-243 | Chakma | 223-274-362-381 | **M74** | 8251-10398-10268-10400 |
| C-244 | Chakma | 183-189-223-362 | **M9a** | 4491-10398-10400-14308 |
| C-245 | Chakma | 182C-189-223-362 | **D5** | 3873-10397-10398-10400 |
| C-246 | Chakma | 223-270-362-381 | **M61** | 10398-10400-12732 |
| C-247 | Chakma | 223-362 | **D4** | 3010-8414-10398-10701-10400 |
| C-248 | Chakma | 111-129-304 | **F1** | 249d-3398-10310-12406 |
| C-249 | Chakma | 129-172-189 | **F1** | 249d-10310-12406 |
| C-250 | Chakma | 223-270-362-381 | **M61** | 10398-10400-12732 |
| C-251 | Chakma | 129-162-172-189-223-304 | **R9b** | 1541-13928C |
| C-252 | Chakma | 129-148-192-223-304 | **R9b** | 1541-13928C |
| C-253 | Chakma | 223-270-319-352 | **M2** | 447G-10398-10400 |
| C-254 | Chakma | 93-192-223-278-362 | **G2a** | 10398-10400-13563 |
| C-255 | Chakma | 129-162-172-304 | **F1** | 249d-10310-12406 |
| C-256 | Chakma | 223-270-301-362 | **M9a** | 4491-10398-10400-14308 |
| C-257 | Chakma | 192-223-278 | **G2a** | 10398-10400-13563 |
| C-257a | Chakma | 189-223-270-278 | **M51** | 150-10398-10400-15317 |
| C-258 | Chakma | 93-192-223-249-319-381 | **M7c3a** | 5442-9824-10398-10400 |
| C-259 | Chakma | 129-162-172-223-270-304 | **R9b** | 1541-13928C |
| C-260 | Chakma | 223-270 | **M** | 10398-10400 |
| C-261 | Chakma | 223-270 | **M** | 10398-10400 |
| C-262 | Chakma | 129-183-189-304 | **F1** | 249d-10310-12406 |
| C-263 | Chakma | 86-223-272-304 | **M7** | 9824-10398-10400 |
| C-264 | Chakma | 86-172-223-362 | **D5** | 3873-10397-10398-10400 |
| C-266 | Chakma | 172-223-362 | **D5** | 3873-10397-10398-10400 |
| C-267 | Chakma | 223-234-311 | **M9a** | 4491-10398-10400-14308 |
| C-268 | Chakma | 223-234-311 | **M9a** | 4491-10398-10400-14308 |
| C-269 | Chakma | 136-223 | **M** | 10398-10400 |
| C-270 | Chakma | 75-93-223-270-274-319 | **M2** | 447G-10398-10400 |
| C-271 | Chakma | 183C-189 | **R** | 15301 |
| C-272 | Chakma | 136-223-301-319 | **M** | 10398-10400 |
| C-273 | Chakma | 129-223-304-311 | **R9b** | 1541-13928C |
| C-274 | Chakma | 223-274-362 | **G3** | 709-10398-10400 |
| C-276 | Chakma | 111-129-304 | **F1** | 249d-3398-10310-12406 |
| C-277 | Chakma | 75-93-129-223-240-248-291 | **M5a** | 709-1888-10398-10400 |
| C-279 | Chakma | 111-129-304 | **F1** | 249d-3398-10310-12406 |
| C-280 | Chakma | 223-311-362 | **M74** | 8251-10398-10268-10400 |
| C-281 | Chakma | 129-223-272 | **M20** | 249d-3200-10398-10400-15691 |
| C-282 | Chakma | 172-223-362 | **D5** | 3873-10397-10398-10400 |
| C-283 | Chakma | 111-129-301-304 | **F1** | 249d-10310-12406 |
| C-284 | Chakma | 111-129-304 | **F1** | 249d-3398-10310-12406 |
| C-285 | Chakma | 172-304 | **F1** | 249d-10310-12406 |
| C-286 | Chakma | 183C-189-223 | **M** | 10398-10400 |
| C-287 | Chakma | 223-311-362 | **M74** | 8251-10398-10268-10400 |
| C-288 | Chakma | 129-223 | **M5** | 1888-10398-10400 |
| C-289 | Chakma | 93-223-290-293C-318T | **M18** | 10398-10400-12007 |
| C-290 | Chakma | 93-223-290-293C-319 | **A11** | 663-9650 |
| C-291 | Chakma | 129-145-301-304-311 | **F1** | 249d-10310-12406 |
| C-292 | Chakma | 223-234 | **M49c** | 3780-10398-10400-10514 |
| C-293- | Chakma | 223-311-362 | **M74** | 8251-10398-10268-10400 |
| C-294 | Chakma | 129-223-264-311 | **M** | 10398-10400 |
| C-295 | Chakma | 129-162-172-304 | **F1** | 249d-10310-12406 |
| C-299 | Chakma | 129-162-172-304 | **F1** | 249d-10310-12406 |
| C-340 | Chakma | 223-304 | **M25** | 10398-10400-15928 |
| C-352 | Chakma | 129-223-297 | **M7b** | 4071-9824-10398-10400 |
| C-359 | Chakma | 129-162-172-304 | **F1** | 249d-10310-12406 |
| C-399 | Chakma | 192-223-249-319-381 | **M7c3a** | 5442-9824-10398-10400 |
| C-399a | Chakma | 192-223-249-319-381 | **M7c3a** | 5442-9824-10398-10400 |
| C-400 | Chakma | 93-192-223-249-319-381 | **M7c3a** | 5442-9824-10398-10400 |
| B-396 | Marma | 129-162-172-293-304 | **F1** | 249d-10310-12406 |
| B-398 | Marma | 93-192-223-249-319-381 | **M7c3a** | 5442-9824-10398-10400 |
| C-313 | Marma | 111-129-304 | **F1** | 249d-8251-10310-12406 |
| M-181 | Marma | 189-223-304-311 | **M25** | 10398-10400-15928 |
| M-182 | Marma | 189-223-227-234-278 | **G2a** | 10398-10400-13563 |
| M-183 | Marma | 129-223-309 | **M** | 10398-10400-12549 |
| M-184 | Marma | 129-136-217-319 | **B4** | 8281-89d |
| M-185 | Marma | 111-129-304-519 | **F1** | 249d-10310-12406 |
| M-292 | Marma | 038-223-292 | **W** | 189-195-204-207-709-12414-15884C |
| M-293 | Marma | 126-223-227-278-362 | **G2a** | 10398-10400-13563 |
| M-294 | Marma | 223-264-311 | **M4'30** | 10398-10400-12007 |
| M-295 | Marma | 129-162-223-304 | **F1** | 249d-10310-12406 |
| M-296 | Marma | 086-129-209-223 | **M20** | 249d-3200-10398-10400-15691 |
| M-297 | Marma | 223-298-319-362 | **M8a** | 8584-10398-10400-14470 |
| M-298 | Marma | 129-209-223-272 | **M20** | 249d-3200-10398-10400-15691 |
| M-302 | Marma | 223-381-390 | **M13** | 152-10398-10400-15924 |
| M-308 | Marma | 86-129-209-223-272 | **M20** | 249d-3200-10398-10400-15691 |
| M-309 | Marma | 189-223-278-362 | **G2a** | 10398-10400-13563 |
| M-310 | Marma | 111-129-304 | **F1** | 249d-8251-10310-12406 |
| M-311 | Marma | 86-223-293-297-390 | **M7b** | 4071-9824-10398-10400 |
| M-312 | Marma | 223-292-297 | **M7b** | 4071-9824-10398-10400 |
| M-314 | Marma | 129-223-311 | **M** | 10398-10400 |
| M-315 | Marma | 129-223-292-293-311 | **M** | 8072-10398-10400 |
| M-316 | Marma | 223-292-297 | **M7b** | 4071-9824-10398-10400 |
| M-317 | Marma | 223-292-297 | **M7b** | 4071-9824-10398-10400 |
| M-318 | Marma | 192-223-249-319-381 | **M7c3a** | 5442-9824-10398-10400 |
| M-320 | Marma | 192-223-249-319-381 | **M7c3a** | 5442-9824-10398-10400 |
| M-321 | Marma | CRS | **U** | 12308-12372 |
| M-322 | Marma | 111-223-235 | **M33c** | 2361-3316-10398-10400 |
| M-323 | Marma | 223-260 | **Z** | 10398-10400-15784 |
| M-324 | Marma | 185-223-260-298 | **Z** | 10398-10400-15784 |
| M-325 | Marma | 185-209-223-260-298-301 | **Z** | 10398-10400-15784 |
| M-326 | Marma | 129-162-172-293-304-519 | **F1** | 249d-10310-12406 |
| M-327 | Marma | 188-223-260-295 | **Z** | 10398-10400-15784 |
| M-328 | Marma | 188-223-288 | **M33** | 2361-10398-10400 |
| M-329 | Marma | 223-298-311-325-327 | **C1** | 249d-8584-10398-10400 |
| M-330 | Marma | 129-145-249-288-301-304-311 | **R22** | 152-329 |
| M-331 | Marma | 168-223-264-311 | **M** | 10398-10400-15049 |
| M-332 | Marma | 223-381-390 | **M13** | 152-10398-10400-15924 |
| M-334 | Marma | 189-223-256-311 | **M10** | 573+C-709-10398-10400 |
| M-335 | Marma | 189-223 | **M13** | 152-3334-10398-10400-15924 |
| M-336 | Marma | 93-223-249-319-381 | **M7c3a** | 5442-9824-10398-10400 |
| M-337 | Marma | 129-145-170-230-249-288-301-304-311 | **R22** | 152-329 |
| M-338 | Marma | 129-145-170-189-249-288-301-304 | **R22** | 152-329 |
| M-339 | Marma | 153-189-223-234-304 | **M49a** | 3780-10398-10400 |
| M-341 | Marma | 223-298-319-362 | **M8a** | 8584-10398-10400-14470 |
| M-343 | Marma | 93-192-223-249-319-381 | **M7c3a** | 5442-9824-10398-10400 |
| M-345 | Marma | 174-223-257-311-362 | **M13** | 152-10398-10400-15924 |
| M-346 | Marma | 129-172-304-519 | **F1** | 249d-10310-12406 |
| M-350 | Marma | 129-172-304 | **F1** | 249d-10310-12406 |
| M-353 | Marma | 189 | **R** | 15301 |
| M-357 | Marma | 129-223-235-297-319 | **M7b** | 4071-9824-10398-10400 |
| M-358 | Marma | 129-168-192-223-249-272-304 | **R9b** | 1541-13928C |
| M-368 | Marma | 16075-93-129-223-240-248-291-399-519 | **M5a** | 709-1888-10398-10400 |
| M-373 | Marma | 223-284-319-362 | **G2a** | 10398-10400-12192-13563 |
| M-378 | Marma | 223-284-319-362 | **G2a** | 10398-10400-12192-13563 |
| M-379 | Marma | 129-162-172-304-519 | **F1** | 249d-10310-12406 |
| M-386 | Marma | 136-223 | **M** | 10398-10400 |
| M-387 | Marma | 223-284-311 | **M60** | 7912-10398-10400 |
| M-389 | Marma | 129-145-170-249-288-301-304-311 | **R22** | 152-329 |
| M-390 | Marma | 223-263-274-311-362 | **G3** | 709-10398-10400 |
| M-392 | Marma | 136-223-311 | **M** | 10398-10400 |
| M-393 | Marma | 223-249-298-319-381 | **M7c3a** | 5442-9824-10398-10400 |
| M-394 | Marma | 51-168-223-264-311 | **M** | 10398-10400-15049 |
| M-395 | Marma | 75-129-162-172-304 | **F1** | 249d-10310-12406 |
| M-53 | Marma | 86-93-218-223-270 | **M** | 10398-10400 |
| M-54 | Marma | 86-129-209-223-272 | **M20** | 249d-3200-10398-10400-15691 |
| M-55 | Marma | 86-93-218-223-278 | **M** | 10398-10400 |
| M-56 | Marma | 223-227-274-278 | **G2a** | 10398-10400-13563 |
| M-57 | Marma | 86-93-218-223-270 | **M** | 10398-10400 |
| M-58 | Marma | 153-223-234 | **M49a** | 3780-8072-10398-10400 |
| M-59 | Marma | 153-223-234 | **M49a** | 3780-8072-10398-10400 |
| M-60 | Marma | 93-168-223-290-319 | **A** | 663 |
| M-61 | Marma | 93-168-223-290-319 | **A** | 663 |
| M-62 | Marma | 189-223-304 | **M25** | 10398-10400-15928 |
| M-63 | Marma | 209-223 | **M20** | 249d-3200-10398-10400-15691 |
| M-64 | Marma | 129-209-223-272 | **M20** | 249d-3200-10398-10400-15691 |
| M-65 | Marma | 129-162-172-304-519 | **F1** | 249d-8390-10310-12406 |
| M-66 | Marma | 129-162-172-304-519 | **F1** | 249d-8390-10310-12406 |
| M-67 | Marma | 129-223-287 | **M47** | 10398-10400 |
| M-68 | Marma | 153-176-223 | **M49a** | 3780-10398-10400 |
| M-69 | Marma | 153-176-223-234 | **M49a** | 3780-10398-10400 |
| M-73 | Marma | 129-153-176-209-223-234-272 | **M20** | 249d-3200-10398-10400-15691 |
| M-79 | Marma | 86-129-209-223-272-319 | **M20** | 249d-3200-10398-10400-15691 |
| M-81 | Marma | 111-129-223-304 | **R9b** | 1541-13928C |
| M-83 | Marma | 86-129-209-223-272 | **M20** | 249d-3200-10398-10400-15691 |
| M-85 | Marma | 86-129-209-223-272 | **M20** | 249d-3200-10398-10400-15691 |
| M-86 | Marma | 86-129-223-272-278 | **M20** | 249d-3200-10398-10400-15691 |
| M-89 | Marma | 223 | **M** | 10398-10400 |
| M-90 | Marma | 223-256 | **M10** | 573+C-709-10398-10400 |
| M-91 | Marma | 93-168-223-311 | **M13** | 152-10398-10400-15924 |
| M-92 | Marma | 129-223-240-248-291 | **M5a** | 709-1888-10398-10400 |
| M-94 | Marma | 86-93-129-209-223-248-272-292 | **M20** | 249d-3200-10398-10400-15691 |
| M-96 | Marma | 86-129-223-272-297 | **M7b** | 4071-9824-10398-10400 |
| M-97 | Marma | 189-223-311 | **M10** | 573+C-709-10398-10400 |
| M-98 | Marma | 129-145-249-288-301-304-311 | **R22** | 152-329 |
| M-99 | Marma | 129-145-249-288-301-304-311 | **R22** | 152-329 |
| B-210 | Tripura | 129-223-249 | **R9b** | 1541-13928C |
| B-211 | Tripura | 172-183C-187-223 | **M75** | 146-150-3316-10398-10400 |
| B-212 | Tripura | 129-223-291 | **M5a** | 709-1888-10398-10400 |
| B-213 | Tripura | 86-223-381-390 | **M13** | 152-10398-10400-15924 |
| B-216a | Tripura | 183C-189 | **R** | 15301 |
| T_313 | Tripura | 223 | **M** | 10398-10400 |
| T-01 | Tripura | 86-129-209-223-272-365 | **M20** | 249d-3200-10398-10400-15691 |
| T-02 | Tripura | 86-129-209-223-272-365 | **M20** | 249d-3200-10398-10400-15691 |
| T-03 | Tripura | 223-297 | **M7b** | 4071-9824-10398-10400 |
| T-04 | Tripura | 136-186-223-304 | **M** | 10398-10400 |
| T-05 | Tripura | 153-223-234-256 | **M49a** | 3780-10398-10400 |
| T-06 | Tripura | 70-223-234 | **M49a** | 3780-10398-10400 |
| T-07 | Tripura | 183-168-223-234 | **M9a** | 4491-10398-10400-14308 |
| T-08 | Tripura | 223-311-362 | **M74** | 8251-10398-10268-10400 |
| T-09 | Tripura | 223-311-362 | **M74** | 8251-10398-10268-10400 |
| T-10 | Tripura | 136-186-223-304 | **M** | 10398-10400 |
| T-101 | Tripura | 136-186-223-304 | **M** | 10398-10400 |
| T-11 | Tripura | 129-162-172-304-519 | **F1** | 249d-8390-10310-12406 |
| T-12 | Tripura | 223-234-497-519 | **M49c** | 3780-10398-10400-10514 |
| T-13 | Tripura | 223-234 | **M49c** | 3780-10398-10400-10514 |
| T-15 | Tripura | 223-235 | **M33c** | 2361-3316-10398-10400 |
| T-16 | Tripura | 16214-223-344 | **M72** | 10398-10400-15644-15820 |
| T-17 | Tripura | 16214-223-344 | **M72** | 10398-10400-15644-15820 |
| T-171 | Tripura | 136-189-223 | **M** | 10398-10400 |
| T-174 | Tripura | 129-162-172-304-327 | **F1** | 249d-8390-10310-12406 |
| T-175 | Tripura | 189-223-256 | **M10** | 573+C-709-10398-10400 |
| T-176 | Tripura | 223-290-319 | **A** | 663-3794 |
| T-179 | Tripura | 223-234-256 | **M49a** | 3780-10398-10400 |
| T-18 | Tripura | 126-223-362 | **M3** | 482-10238-10398-10400 |
| T-19 | Tripura | 126-223-344-519 | **M3** | 482-10238-10398-10400 |
| T-20 | Tripura | 126-223-344-519 | **M3** | 482-10238-10398-10400 |
| T-21 | Tripura | 129-223-249 | **R9b** | 1541-13928C |
| T-22 | Tripura | 183-189-249-304-311 | **R22** | 152-329 |
| T-23 | Tripura | 126-223-344-519 | **M3** | 482-10238-10398-10400 |
| T-24 | Tripura | 183C-189-223 | **M13** | 152-10398-10400-15924 |
| T-25 | Tripura | 129-172-189-304 | **F1** | 249d-3780-10310-12406 |
| T-27 | Tripura | 129-172-189-304 | **F1** | 249d-3780-10310-12406 |
| T-28 | Tripura | 129-172-189-304 | **F1** | 249d-3780-10310-12406 |
| T-29 | Tripura | 129-172-189-304 | **F1** | 249d-3780-10310-12406 |
| T-30 | Tripura | 111-129-304-519 | **F1** | 249d-10310-12406 |
| T-303 | Tripura | 111-129-304 | **F1** | 249d-10310-10454-12406 |
| T-304 | Tripura | 111-168-172 | **R31a** | 146-338-15884 |
| T-305 | Tripura | 129-162-172-304 | **F1** | 249d-10310-12406 |
| T-306 | Tripura | 172-183C-223-311-381-390 | **E** | 3027-10398-10370-10400 |
| T-307 | Tripura | 93-192-223-249-319-381 | **M7c3a** | 5442-9824-10398-10400 |
| T-308 | Tripura | 192-223-319-381 | **M7c3a** | 5442-9824-10398-10400 |
| T-32 | Tripura | 129-145-249-288-301-304-311 | **R22** | 152-329 |
| T-328 | Tripura | 111-168-183-172-189 | **R31a** | 146-338-15884 |
| T-33 | Tripura | 111-129-249-288-301-304 | **R22** | 152-329 |
| T-333 | Tripura | 189-223 | **M13** | 152-3334-10398-10400-15924 |
| T-34 | Tripura | 223 | **M** | 10398-10400 |
| T-342 | Tripura | 93-192-223-249-319-381 | **M7c3a** | 5442-9824-10398-10400 |
| T-343 | Tripura | 93-192-223-249-319-381 | **M7c3a** | 5442-9824-10398-10400 |
| T-344 | Tripura | 193-223-291 | **N21** | 150-337d-8701 |
| T-347 | Tripura | 147-189-223 | **M62** | 150-3511-8063-10398-10400 |
| T-348 | Tripura | 70-223-234-304 | **M49a** | 3780-10398-10400 |
| T-349 | Tripura | 126-223-362 | **M3** | 482-10238-10398-10400 |
| T-35 | Tripura | 111-129-304-519 | **F1** | 249d-10310-12406 |
| T-351 | Tripura | 223-324-362 | **M9a** | 4491-10398-10400-14308 |
| T-354 | Tripura | 129-309-318T-519 | **U7** | 980-12308-12372 |
| T-355 | Tripura | 129-304-311 | **F1** | 249d-10310-12406 |
| T-356 | Tripura | 129-309-318T-519 | **U7** | 980-12308-12372 |
| T-36 | Tripura | 129-192-223 | **R9b** | 1541-13928C |
| T-361 | Tripura | 136-223 | **M** | 3585-10398-10400 |
| T-362 | Tripura | 111-129-304 | **F1** | 249d-10310-10454-12406 |
| T-363 | Tripura | 86-223-381-390 | **M13** | 152-10398-10400-15924 |
| T-364 | Tripura | 93-223-234-390 | **M49a** | 3780-10398-10400 |
| T-365 | Tripura | 223-298-319-362 | **M8a** | 8584-10398-10400-14470 |
| T-366 | Tripura | 136-223 | **M** | 3585-10398-10400 |
| T-367 | Tripura | 129-172-189-304 | **F1** | 249d-10310-12406 |
| T-369 | Tripura | 223-324-362 | **M9a** | 4491-10398-10400-14308 |
| T-37 | Tripura | 129-223 | **M5** | 1888-10398-10400 |
| T-371 | Tripura | 129-162-172-304-327-519 | **F1** | 249d-10310-12406 |
| T-372 | Tripura | 70-223-234-497 | **M49a** | 3780-10398-10400 |
| T-374 | Tripura | 51-153-223-234-272 | **M49a** | 3780-10398-10400 |
| T-375 | Tripura | 223-311-362 | **M74** | 8251-10398-10268-10400 |
| T-376 | Tripura | 304-311 | **F1** | 249d-10310-12406 |
| T-377 | Tripura | 129-162-172-304 | **F1** | 249d-10310-12406 |
| T-379 | Tripura | 93-223-225-390 | **M48** | 10398-10400-10684-15900 |
| T-38 | Tripura | 148-192-223 | **M** | 10398-10400 |
| T-380 | Tripura | 129-162-172-304 | **F1** | 249d-10310-12406 |
| T-381 | Tripura | 192-223-249-319-381 | **M7c3a** | 5442-9824-10398-10400 |
| T-382 | Tripura | 129-162-172-304-309 | **F1** | 249d-10310-12406 |
| T-383 | Tripura | 136-223 | **M** | 3585-10398-10400 |
| T-385 | Tripura | 136-223 | **M** | 3585-10398-10400 |
| T-39 | Tripura | 129-192-311 | **R3** | 150-241 |
| T-391 | Tripura | 111-129-304-519 | **F1** | 249d-10310-12406 |
| T-395 | Tripura | 129-304 | **F1** | 249d-10310-12406 |
| T-40 | Tripura | 223-234-318T | **M18** | 10398-10400-12007 |
| T-42 | Tripura | 183C-189-304 | **F1** | 249d-10310-12406 |
| T-43 | Tripura | 129-223-272 | **M20** | 249d-3200-10398-10400-15691 |
| T-44 | Tripura | 223-274-362 | **G3** | 709-10398-10400 |
| T-45 | Tripura | 111-129-304 | **F1** | 249d-10310-10454-12406 |
| T-46 | Tripura | 148-192-223 | **M** | 10398-10400 |
| T-50 | Tripura | 129-162-189-272-293-304 | **F1** | 249d-10310-12406 |
| T-51 | Tripura | 86-172-223-304-381-390 | **E** | 3027-10398-10400 |
| T-52 | Tripura | 038-192-223-234 | **M49a** | 3780-10398-10400 |
