# Supplementary material for: Genetic Structure of Tibeto-Burman Populations of Bangladesh: Evaluating the Gene Flow along the Sides of Bay-of-Bengal
Source: PLoS One. 2013 Oct 9;8(10):e75064. doi: 10.1371/journal.pone.0075064 (PMC3794028; doi:10.1371/journal.pone.0075064)
Supplement: Table S2 — Y-chromosome age estimates for haplogroups O2a and O3a3c among population groups of India E/SE Asia and Bangladesh. (DOC) [file pone.0075064.s005.doc]

|  | **O2a (M95)** | | | **O3a3c (M134)** | | |
| --- | --- | --- | --- | --- | --- | --- |
| **Group** | **Sample size (*n*)** | **Variance** | **Age (kya)** | **Sample size (*n*)** | **Variance** | **Age (kya)** |
| India (Overall) | 178 | 0.36 | 15.9 ± 1.6 | 178 | 0.26 | 10.4 ± 3.2 |
| Southeast Asia (Overall) | 142 | 0.52 | 22.4 ± 4.9 | 142 | 0.50 | 19.5 ± 5.3 |
| Bangladesh (Overall) | 25 | 0.38 | 16.2+2 | 45 | 0.27 | 12.8+1.7 |
| Chakma | 6 | 0.37 | 12.7+1.5 | 16 | 0.28 | 14.2+2.8 |
| Marma | 9 | 0.35 | 15.8+2.7 | 14 | 0.23 | 9.7+1.1 |
| Tripura | 10 | 0.38 | 15.2+2.5 | 15 | 0.24 | 10.6+1.8 |

Age estimates obtained using the TD statistic, assuming a mutation rate of 6.9 x 10^-4^ (Zhivotovsky et al. 2004), based on variation at 14 and 7 common Y-STR loci for haplogroup O2a and O3a3c respectively.
