# Supplementary material for: Genetic Structure of Tibeto-Burman Populations of Bangladesh: Evaluating the Gene Flow along the Sides of Bay-of-Bengal
Source: PLoS One. 2013 Oct 9;8(10):e75064. doi: 10.1371/journal.pone.0075064 (PMC3794028; doi:10.1371/journal.pone.0075064)
Supplement: Table S3 — Y STR haplotype data of the studied populations. (DOC) [file pone.0075064.s006.doc]

| **Population** | **Sample no** | **HG** | **DYS19** | **DYS389ab** | **DYS389cd** | **DYS390** | **DYS391** | **DYS392** | **DYS393** | **DYS437** | **DYS438** | **DYS439** | **DYS448** | **DYS456** | **DYS458** | **DYS635** | **GATA_H4** |
| --- | --- | --- | --- | --- | --- | --- | --- | --- | --- | --- | --- | --- | --- | --- | --- | --- | --- |
| Chakma | CB211 | N1 | 14 | 15 | 12 | 23 | 10 | 14 | 13 | 16 | 11 | 12 | 19 | 15 | 18 | 22 | 12 |
| Chakma | CB230 | N1 | 15 | 15 | 13 | 23 | 10 | 14 | 12 | 14 | 10 | 12 | 20 | 15 | 17 | 22 | 11 |
| Chakma | CB277 | N1 | 14 | 16 | 13 | 23 | 10 | 14 | 13 | 15 | 11 | 12 | 20 | 15 | 18 | 20 | 12 |
| Chakma | CB231 | N1 | 14 | 16 | 13 | 24 | 11 | 14 | 12 | 16 | 12 | 12 | 20 | 14 | 18 | 22 | 12 |
| Tripura | T394 | N1 | 14 | 16 | 12 | 24 | 10 | 14 | 13 | 15 | 11 | 12 | 21 | 15 | 18 | 20 | 11 |
| Tripura | T395 | N1 | 14 | 16 | 13 | 23 | 10 | 14 | 13 | 15 | 11 | 12 | 20 | 15 | 18 | 22 | 12 |
| Chakma | CB233 | NO | 14 | 16 | 13 | 24 | 10 | 14 | 11 | 15 | 11 | 11 | 20 | 15 | 18 | 20 | 12 |
| Marma | MB334 | NO | 14 | 16 | 12 | 23 | 9 | 14 | 12 | 14 | 11 | 12 | 20 | 15 | 19 | 21 | 12 |
| Tripura | T392 | NO | 14 | 16 | 13 | 25 | 10 | 15 | 11 | 15 | 12 | 12 | 20 | 15 | 19 | 20 | 11 |
| Chakma | CB232 | O2a | 14 | 15 | 14 | 24 | 10 | 14 | 13 | 14 | 10 | 12 | 20 | 16 | 17 | 22 | 13 |
| Chakma | CB237 | O2a | 15 | 16 | 13 | 24 | 10 | 14 | 12 | 15 | 11 | 11 | 20 | 15 | 18 | 20 | 12 |
| Chakma | CB252 | O2a | 15 | 17 | 13 | 25 | 10 | 14 | 12 | 14 | 11 | 11 | 20 | 17 | 16 | 22 | 13 |
| Chakma | CB255 | O2a | 14 | 16 | 13 | 23 | 10 | 14 | 13 | 14 | 11 | 12 | 19 | 16 | 16 | 21 | 13 |
| Chakma | CB270 | O2a | 14 | 16 | 12 | 23 | 11 | 14 | 12 | 15 | 11 | 12 | 20 | 15 | 17 | 20 | 12 |
| Chakma | CB281 | O2a | 15 | 16 | 13 | 24 | 10 | 14 | 13 | 14 | 10 | 12 | 19 | 16 | 16 | 21 | 13 |
| Marma | MB183 | O2a | 14 | 15 | 13 | 25 | 10 | 14 | 12 | 14 | 11 | 11 | 19 | 14 | 17 | 22 | 12 |
| Marma | MB310 | O2a | 14 | 16 | 12 | 23 | 10 | 14 | 12 | 15 | 11 | 12 | 20 | 15 | 18 | 20 | 12 |
| Marma | MB325 | O2a | 14 | 16 | 13 | 24 | 10 | 14 | 13 | 14 | 10 | 11 | 18 | 15 | 16 | 20 | 11 |
| Marma | MB327 | O2a | 15 | 16 | 13 | 24 | 10 | 14 | 13 | 14 | 10 | 11 | 20 | 15 | 16 | 22 | 11 |
| Marma | MB390 | O2a | 14 | 16 | 12 | 23 | 11 | 14 | 13 | 15 | 11 | 12 | 19 | 15 | 16 | 21 | 13 |
| Marma | MB50 | O2a | 14 | 16 | 13 | 23 | 11 | 14 | 12 | 15 | 11 | 12 | 20 | 16 | 17 | 20 | 12 |
| Marma | MB51 | O2a | 14 | 16 | 12 | 24 | 10 | 14 | 12 | 15 | 11 | 11 | 19 | 16 | 18 | 21 | 11 |
| Marma | MB85 | O2a | 14 | 16 | 12 | 24 | 10 | 15 | 12 | 15 | 11 | 12 | 20 | 15 | 18 | 20 | 12 |
| Marma | MB98 | O2a | 14 | 16 | 12 | 23 | 10 | 14 | 13 | 14 | 11 | 12 | 20 | 15 | 18 | 20 | 12 |
| Tripura | T11 | O2a | 15 | 15 | 13 | 23 | 10 | 14 | 11 | 14 | 10 | 12 | 19 | 15 | 17 | 21 | 11 |
| Tripura | T18 | O2a | 14 | 16 | 12 | 23 | 10 | 15 | 11 | 15 | 11 | 11 | 20 | 15 | 18 | 20 | 12 |
| Tripura | T23 | O2a | 14 | 16 | 12 | 24 | 11 | 14 | 11 | 15 | 11 | 12 | 20 | 15 | 18 | 20 | 12 |
| Tripura | T299 | O2a | 15 | 16 | 13 | 24 | 10 | 14 | 11 | 14 | 10 | 11 | 18 | 15 | 17 | 20 | 11 |
| Tripura | T303 | O2a | 14 | 16 | 12 | 23 | 10 | 14 | 12 | 15 | 11 | 12 | 20 | 16 | 17 | 20 | 12 |
| Tripura | T306 | O2a | 14 | 16 | 12 | 23 | 10 | 14 | 12 | 14 | 11 | 12 | 20 | 15 | 19 | 21 | 12 |
| Tripura | T360 | O2a | 14 | 16 | 13 | 23 | 11 | 14 | 12 | 14 | 11 | 12 | 20 | 16 | 17 | 21 | 12 |
| Tripura | T361 | O2a | 14 | 16 | 12 | 25 | 11 | 14 | 11 | 15 | 11 | 12 | 20 | 15 | 19 | 20 | 12 |
| Tripura | T380 | O2a | 15 | 16 | 13 | 24 | 10 | 14 | 12 | 14 | 10 | 12 | 19 | 16 | 16 | 22 | 13 |
| Tripura | T390 | O2a | 14 | 15 | 13 | 24 | 11 | 14 | 13 | 14 | 10 | 12 | 19 | 15 | 16 | 22 | 12 |
| Chakma | CB100 | O3 | 15 | 15 | 13 | 24 | 10 | 13 | 11 | 15 | 11 | 12 | 20 | 15 | 18 | 23 | 12 |
| Chakma | CB193a | O3 | 15 | 15 | 12 | 24 | 10 | 14 | 11 | 15 | 12 | 12 | 20 | 15 | 18 | 21 | 12 |
| Chakma | CB202 | O3 | 15 | 15 | 13 | 24 | 10 | 13 | 13 | 14 | 10 | 12 | 18 | 16 | 16 | 23 | 13 |
| Chakma | CB205 | O3 | 14 | 15 | 13 | 24 | 10 | 14 | 12 | 15 | 11 | 12 | 20 | 15 | 18 | 21 | 12 |
| Chakma | CB220 | O3 | 14 | 15 | 13 | 23 | 10 | 14 | 11 | 15 | 11 | 11 | 21 | 15 | 19 | 22 | 11 |
| Chakma | CB225 | O3 | 14 | 16 | 13 | 24 | 10 | 13 | 11 | 15 | 11 | 12 | 20 | 16 | 18 | 21 | 12 |
| Chakma | CB242 | O3 | 15 | 16 | 13 | 23 | 10 | 13 | 12 | 14 | 10 | 11 | 21 | 15 | 16 | 21 | 11 |
| Chakma | CB253 | O3 | 14 | 16 | 12 | 23 | 10 | 13 | 13 | 14 | 12 | 12 | 19 | 15 | 17 | 21 | 11 |
| Chakma | CB260 | O3 | 14 | 16 | 12 | 24 | 10 | 14 | 11 | 15 | 11 | 12 | 20 | 15 | 18 | 21 | 12 |
| Chakma | CB275 | O3 | 14 | 16 | 13 | 24 | 10 | 13 | 12 | 14 | 11 | 11 | 20 | 16 | 16 | 22 | 12 |
| Chakma | CB193 | O3 | 15 | 16 | 12 | 23 | 10 | 14 | 12 | 15 | 11 | 12 | 19 | 16 | 18 | 21 | 12 |
| Chakma | CB218 | O3 | 14 | 16 | 13 | 24 | 10 | 14 | 11 | 15 | 11 | 12 | 20 | 15 | 18 | 21 | 12 |
| Chakma | CB227 | O3 | 14 | 15 | 13 | 25 | 10 | 14 | 11 | 16 | 11 | 13 | 20 | 16 | 18 | 21 | 12 |
| Chakma | CB236 | O3 | 15 | 15 | 12 | 24 | 10 | 14 | 12 | 15 | 11 | 12 | 20 | 16 | 18 | 21 | 12 |
| Chakma | CB285 | O3 | 14 | 16 | 12 | 23 | 10 | 14 | 11 | 15 | 11 | 11 | 20 | 15 | 19 | 22 | 12 |
| Chakma | CB400 | O3 | 14 | 16 | 13 | 24 | 10 | 14 | 11 | 15 | 11 | 11 | 20 | 15 | 18 | 21 | 12 |
| Marma | MB182 | O3 | 14 | 16 | 13 | 23 | 10 | 14 | 11 | 15 | 11 | 11 | 20 | 15 | 18 | 20 | 12 |
| Marma | MB297 | O3 | 14 | 16 | 12 | 23 | 10 | 14 | 11 | 15 | 11 | 11 | 19 | 15 | 18 | 21 | 12 |
| Marma | MB335 | O3 | 14 | 16 | 12 | 23 | 10 | 13 | 11 | 14 | 10 | 12 | 19 | 15 | 17 | 20 | 11 |
| Marma | MB55 | O3 | 14 | 16 | 12 | 23 | 10 | 13 | 11 | 16 | 11 | 12 | 20 | 15 | 18 | 20 | 11 |
| Marma | MB57 | O3 | 14 | 17 | 13 | 23 | 10 | 13 | 12 | 16 | 11 | 12 | 19 | 15 | 18 | 20 | 11 |
| Marma | MB59 | O3 | 14 | 16 | 12 | 24 | 10 | 14 | 12 | 14 | 11 | 12 | 20 | 15 | 18 | 22 | 12 |
| Marma | MB61 | O3 | 14 | 15 | 12 | 23 | 11 | 14 | 11 | 15 | 11 | 12 | 19 | 15 | 18 | 20 | 12 |
| Marma | MB67 | O3 | 14 | 16 | 12 | 24 | 10 | 14 | 11 | 15 | 11 | 11 | 19 | 15 | 18 | 20 | 12 |
| Marma | MB68 | O3 | 14 | 16 | 13 | 23 | 10 | 14 | 12 | 15 | 11 | 12 | 20 | 15 | 17 | 22 | 11 |
| Marma | MB69 | O3 | 15 | 16 | 13 | 23 | 10 | 14 | 11 | 16 | 11 | 11 | 19 | 15 | 16 | 22 | 12 |
| Marma | MB79 | O3 | 15 | 15 | 13 | 22 | 10 | 14 | 11 | 14 | 10 | 12 | 19 | 15 | 16 | 21 | 11 |
| Marma | MB81 | O3 | 14 | 17 | 12 | 23 | 10 | 14 | 11 | 15 | 11 | 12 | 19 | 15 | 18 | 20 | 12 |
| Marma | MB94 | O3 | 14 | 16 | 12 | 23 | 11 | 13 | 11 | 15 | 11 | 12 | 18 | 15 | 18 | 20 | 12 |
| Marma | MB99 | O3 | 15 | 16 | 13 | 22 | 10 | 14 | 11 | 14 | 10 | 12 | 19 | 14 | 17 | 20 | 12 |
| Tripura | T12 | O3 | 14 | 16 | 12 | 23 | 10 | 14 | 11 | 15 | 11 | 11 | 20 | 17 | 19 | 20 | 12 |
| Tripura | T15 | O3 | 14 | 16 | 12 | 23 | 10 | 14 | 11 | 15 | 11 | 11 | 20 | 17 | 19 | 20 | 12 |
| Tripura | T16 | O3 | 14 | 16 | 12 | 23 | 10 | 14 | 11 | 15 | 11 | 12 | 20 | 17 | 19 | 20 | 12 |
| Tripura | T171 | O3 | 14 | 16 | 13 | 24 | 11 | 14 | 11 | 14 | 11 | 12 | 17 | 15 | 18 | 21 | 11 |
| Tripura | T27 | O3 | 14 | 17 | 12 | 24 | 11 | 14 | 11 | 15 | 11 | 12 | 19 | 15 | 18 | 20 | 12 |
| Tripura | T29 | O3 | 14 | 17 | 12 | 24 | 11 | 14 | 11 | 15 | 11 | 12 | 19 | 15 | 18 | 20 | 12 |
| Tripura | T3 | O3 | 14 | 16 | 12 | 23 | 11 | 14 | 11 | 14 | 11 | 11 | 20 | 16 | 18 | 20 | 12 |
| Tripura | T30 | O3 | 14 | 15 | 12 | 23 | 10 | 14 | 11 | 15 | 11 | 12 | 20 | 15 | 20 | 20 | 12 |
| Tripura | T347 | O3 | 14 | 16 | 12 | 24 | 10 | 14 | 11 | 15 | 11 | 12 | 20 | 15 | 20 | 21 | 12 |
| Tripura | T316 | O3 | 15 | 15 | 13 | 24 | 10 | 14 | 12 | 14 | 10 | 12 | 20 | 15 | 18 | 22 | 11 |
| Tripura | T330 | O3 | 14 | 16 | 13 | 23 | 10 | 13 | 11 | 15 | 11 | 12 | 20 | 15 | 18 | 20 | 12 |
| Tripura | T363 | O3 | 15 | 16 | 12 | 24 | 10 | 14 | 11 | 15 | 11 | 11 | 20 | 15 | 18 | 20 | 12 |
| Tripura | T368 | O3 | 15 | 17 | 13 | 22 | 10 | 13 | 11 | 14 | 10 | 12 | 19 | 14 | 18 | 21 | 12 |
| Tripura | T40 | O3 | 14 | 15 | 12 | 23 | 10 | 14 | 11 | 15 | 11 | 12 | 20 | 15 | 20 | 20 | 12 |
| Tripura | T10 | O3 | 15 | 16 | 13 | 24 | 10 | 13 | 11 | 16 | 12 | 12 | 21 | 15 | 19 | 20 | 12 |
